# Investigating the existence of social networks in cheating behaviors in medical students

**DOI:** 10.1186/s12909-018-1299-7

**Published:** 2018-08-09

**Authors:** Jorge Monteiro, Fernanda Silva-Pereira, Milton Severo

**Affiliations:** 1 Departamento de Ciências da Saúde Pública e Forenses e Educação Médica, Unidade de Educação Médica, Piso 6, Faculdade de Medicina da Universidade do Porto, Al. Prof. Hernâni Monteiro, 4200-319 Porto, Portugal; 2 EPIUnit – Instituto de Saúde Pública, Universidade do Porto, Porto, Portugal

**Keywords:** Academic dishonesty, Undergraduate, Cheating, Social network, Assessment

## Abstract

**Background:**

Most studies on academic cheating rely on self-reported questionnaires and focus on the individual, overlooking cheating as a group activity. The aim of this study is to estimate the true prevalence of cheating/anomalies among medical students using a statistical index developed for this purpose, and to explore the existence of social networks between anomalies in students’ results.

**Methods:**

Angoff’s *A* index was applied to a sample of 30 written examinations, with a total of 1487 students and 7403 examinations taken, from the 2014/2015 academic year of the Faculty of Medicine of the University of Porto to detect anomaly pairs. All analyses are within the same academic year and not across years. Through simulations, the sensitivity and specificity of the statistical method was determined, and the true prevalence of anomalies/cheating was estimated. Networks of anomaly pairs were created to search for patterns and to calculate their density.

**Results:**

The percentage of students who cheated at least once increased with the year of medical school, being lowest in the first year (3.4%) and highest in the fifth (17.3%). The year of medical school was associated with anomalies (*p* < 0.05). The network’s density was also lowest in the first year (1.12E-04) and highest in the fifth (8.20E-04). The true prevalence of anomalies was estimated to be 1.85% (95%CI: 1.07–3.20%).

**Conclusions:**

These findings suggest that some students are involved in social networks of cheating, which grow over time, resulting in an increase of anomalies/cheating in later academic years.

**Electronic supplementary material:**

The online version of this article (10.1186/s12909-018-1299-7) contains supplementary material, which is available to authorized users.

## Background

Recent studies have shown the importance of social networks in medical education [1–3]. The main findings are that friends are usually similar in demographic factors, and attitudes and behaviors may result from peer influence. Also, a recent study showed that friendship has an impact on students’ grades and the most common argument presented is that friendship improves teamwork and consequently learning [4] in medical students. A less benign explanation is that similarity between grades may be partly explained by cheating. Đogaš et al. showed that over 80% of medical students would ask family, friends, colleagues or even strangers to copy test answers during an examination [5] and engagement with others in academic cheating among medical students was independent of nationality [6]. Several studies have determined the prevalence of academic cheating [7–10]. These are mostly surveys that were conducted in medical schools, relying on students’ perception and self-admittance of such practices [11]. The reported prevalence of cheating among medical students in the United States (US) ranges from 5 to 58%. When considering only cheating in written examinations, studies completed in the United Kingdom and the US reported a prevalence of 1 to 3% [9, 12], while studies conducted in Canada, India, Pakistan and Croatia have a self-reported prevalence of 32 to 55% [13–16].

Considering this high prevalence of cheating, it should not be studied as an individual phenomenon but as a possible social network phenomenon. Where the social network of cheating is defined as a phenomenon where groups of students collude in advance to copy answers between them during examinations.

A recent study showed that prior observation of unprofessional behavior is connected with later participation in unprofessional behavior [17], thus peer influence may exist. It has also been shown that unprofessional behavior during medical school is associated with unprofessional behavior and a higher rate of disciplinary actions during medical practice [4].

Some variables have been identified as risk factors for cheating [18–21]. In a study conducted in Canada, students were asked their opinion on which factors affected the decision for a student to cheat. The main reasons for cheating were low instructor vigilance, unfair examinations, an instructor who did not care about cheating, and dependence of financial support and long-term goals on good grades; while high instructor vigilance, fair examinations, high punishment for getting caught, essay examinations, widely spaced seating in examinations and valuable course material were considered factors that decrease cheating [13].

Several statistical methods have been developed to detect anomalies in multiple-choice examinations [22–25]. These methods calculate the likelihood of matching correct or/and incorrect response alternatives between two examinees, and if the likelihood is low enough, the null hypothesis (that no one cheated) can be rejected.

Despite several different methods to detect copying answers on multiple-choice examinations, it is not clear which one is the best at detecting true cheaters. Only those that have been previously tested against other indices are considered here. Angoff et al. compared eight indices using real data [22] and his *A* index is of particular interest, and the one used in the computer program Acinonyx [23]. Wollak et al. compared the K, Scrutiny!, g2 and ω indices using real data, and ω had the most power to detect true cheaters [24]. Zopluoglu and Davenport compared the ω and the generalized binominal test (GBT) indices using simulated data, where the GBT index performed slightly better [25]. However, both the ω and GBT indices rely on the item response theory, and the item parameters have to be estimated before these indices can be calculated.

All these methods detect undue similarity/anomaly between students and this can result from many factors other than cheating, as an example, it may result from students who study together as they may present a very similar answer pattern.

From now on when we write anomaly we are referring to statistical exceptions which are unexplained because they are outside the normal bounds of chance.

Our purpose is to apply a statistical method in written medical examinations to detect cheating/anomaly pairs, and then create a network with the students detected and the connections between them to detect patterns and the possible effect of a social network in cheating. A secondary objective is to estimate the true prevalence of anomalies, and to determine which factors are associated with anomalies.

## Methods

Data consisted of a sample of written examinations from the 2014/2015 academic year at the Faculty of Medicine of the University of Porto (FMUP). Examinations of the regular phase from both semesters from the first to the fifth year were included. Thirty courses, five from the first year, eight from the second, seven from the third, five from the fourth and five from the fifth were included in this study, for a total of 7403 examinations from 1487 examinees. Each course had an average of 247 examinees, with the highest being 325 and the lowest 126. Students in the fourth and the fifth year are divided into clinical rotations, and examinations are administrated at different occasions. In these, the mean number of examinees was 57. The use or presence of cell phones was not permitted during examinations, thus it was not possible to use cell phones to exchange information.

We used a version of Angoff’s *A* index to detect anomaly pairs [22]. This index is the same that is used in the computer program Acinonyx [23], but we replaced the Bonferroni correction with the Šidák correction [26] to control the familywise error rate. Thus, let R_i_ represent the number of correct answers by examinee i, R_j_ the number of correct answers by examinee j and R_ij_ the number of correct answers shared by both. The likelihood of agreement between i and j is determined by calculating the residual of R_ij_ after regression on √(R_i_.R_j_) and R_ij_. The significance level was set at 0.05. For N number of examinees, N(N − 1)/2 pairs were tested. First, we calculated an uncorrected probability, P_raw_, which was then adjusted for the inflation of error type I by the Šidák correction given the corrected probability, P_correct_. The P_correct_ is equal to 1- (1-p)^1/ (N(N − 1)/2)^.

As students in the fourth and the fifth year were divided into clinical rotations, and the courses have different test forms at different time periods (meaning that students with tests at different times cannot cheat from each other), the sample size was too small to obtain a reliable estimate of the item parameters for every test form in order to use the ω and the GBT indices.

To determine the sensitivity (SEN) and specificity (SPE) of this method, we simulated a series of examinations considering similar parameterization as the study by McManus [23]. These had 50 or 100 items with 5 possible responses, and were composed of 300 examinees. The probability of a student answering correctly to an item was determined by ${p}_i\left(\theta \right)={c}_i+\frac{1-{c}_i}{1+{e}^{-{a}_i\left(\theta -{b}_i\right)}}$, with ability *θ* normally distributed with a mean of zero and a standard deviation of 1, the discrimination patterns *a* normally distributed with a mean of 0.31 and a standard deviation of 0.15, the difficulty parameter *b* uniformly distributed from − 3 to 3 and a guessing probability *c* normally distributed with a mean of 0.2 and a standard deviation of 0.025. A 10% rate of copying was used, where 10% of examinees with below average ability (theta < 0) would copy a percentage of items from a stronger examinee (theta > 0). The percentage of items copied was manipulated in 7 different levels from 70 to 100, in increments of 5. Fifty examinations were simulated for each level, and sensitivity and specificity were calculated.

To estimate the real amount of anomalies in each written examination, we adjusted the number of anomaly pairs detected to the sensitivity and specificity that were associated with the amount of anomalies that we were considering (between 70 and 100%). When we applied the *A* index, it told us which students were classified as having similar response patterns (positive test). The percentage of those students is referred to as the apparent prevalence (AP) and is obtained by

$$ AP=\frac{\# positives}{\# total\ students}. $$

We want to know the percentage of students who are truly similar, the true prevalence (TP)

$$ TP=\frac{\# cheaters}{\# total\ students}. $$

The following approach can be used to estimate the TP using the following relationship with the AP and taking into account the SEN and SPE of the *A* index [27].

$$ AP= TP\times SEN+\left(1- TP\right)\times \left(1- SPE\right). $$

To obtain the TP, we used the SEN and the SPE computed in the simulation study. The true percentage of anomalies/cheating was estimated by (AP + SPE - 1) / (SEN + SPE -1) [28]. For exams with less than 75 items, we used the SEN and the SPE determined for 50 items, and in those with more than 75 items, we used the SEN and the SPE determined for 100 items. A Bayesian approach was used to estimate the TP [27].

We evaluated the effects of the year of medical school, semester, length of examination, use of alternate test forms, number of multiple choice options, mean percentage of correct answers and mean percentage of missing answers on the AP using independent sample t-test and ANOVA. Considering the AP had a skewed distribution, a log transformation was used and the respective results were presented using geometric mean and geometric standard deviation. The adjusted geometric means and respective 95% confidence intervals (95%CIs) were estimated using linear regression.

The network diagram was constructed in the following manner: red point (edge) means that the student has at least one anomaly identified, a line leading from one student to another student indicates that the two students were identified in one examination as a cheating/anomaly pair. All students that were not identified at least in one anomaly are not represented in the network diagram. The following two measures of social network were used: Network Density and cliques. Network Density is the number of actual connections between the students divided by the number of possible connections. Network Density values range from 0 to 1, where higher values means a greater degree of interaction between the students detected. A clique is the maximum number of edges (students), where all possible connections are present among themselves.

To construct and analyze the networks of anomaly pairs, we used the network and igraph package for R [29].

Our study only used administrative (secondary) data collected by an optical mark and character reading software that recodes and corrects after an examination. The students know by effects of correction of the examinations that teachers can always ask for similarity between the students´ answers. As such, the consent of study subjects was presumed. Nevertheless, the data was anonymised before release to researchers, all direct identifiers such names, student numbers and course identifiers were removed. As a secondary analysis of this study, we pretended to analyse if students were seated together or not, and in that case, another researcher was asked to look at the set of pairs that included the detected pairs and the set of random pairs in order to diminish the probability of re-identification. The present study did not intend to prejudice any of its students by the use of its conclusions. This research design did not adversely affect students’ educational progress or students’ well-being. Nevertheless, the Centro Hospitalar São João Ethics Committee for Health (CES) was consulted before the realization of the study and considered that approval by the CES was not required.

## Results

### Anomalies/cheating prevalence and the effect of variables

**Fig. 1:**
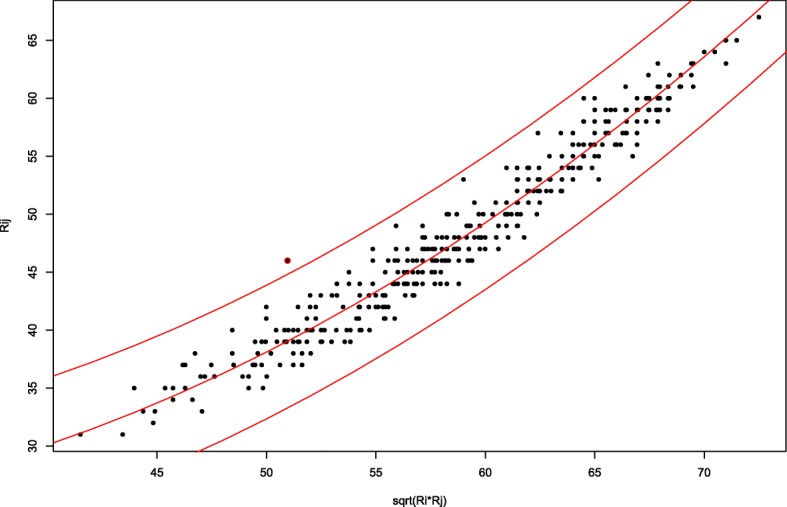
Plot of all pairs of students Rij against the square root(RiRj) from one examination. The red line indicates the mean and the thresholds where the similarity equals the significance level with the correction of Šidák

Figure 1 shows the detection of one pair that shows undue similarity/anomaly in one examination according to the *A* index.

The *A* index detected 151 pairs with a median P_corrected_ of 1.5 × 10^− 6^, a first quartile of 3.3 × 10^− 8^ and a third quartile of 1.5 × 10^− 5^. The pair detected with maximum P_corrected_ was 0.003. For two courses where we detected pairs, we had the seating plans, and we confirmed that the seven pairs detected were seated in adjacent seats. Also, 13 of the courses usually sat their students alphabetically in the examination room according to the given name, and within the 63 anomaly pairs detected in these courses, 12 (19%) pairs had the same given name, 13 (21%) pairs had the same first letter of the given name and 17 (26.9%) pairs differed only in 1 or 2 letters in the first letter of the given name.

Additional file 1: Table S1, shows the distribution of anomalies among all courses included in this study. The geometric mean AP of anomalies detected was 2.18% (95%CI: 1.43–3.19%). When adjusting for SEN and SPE of the method, the TP of anomalies was 1.85% (95%CI: 1.07–3.20%). In the course with the highest amount of apparent anomalies, 11.0% of the students were detected anomalies, while in 5 courses, no anomalies were found (see Additional file 1: Table S1).

The association between apparent anomalies and the variables tested are shown in Table 1.

**Table 1:** Factors associated with apparent prevalence of anomalies

	N	GM (GSD)	*p*-value	Adjusted GM (95%CI)^a^	*p*-value
Year					
1	5	0.5% (2.28)	0.049	0.6% (0.0–1.7%)	0.066
2	8	2.0% (2.09)		2.3% (1.1–4.2%)	
3	7	3.7% (1.41)		2.5% (1.0–5.2%)	
4	5	2.3% (2.45)		3.0% (1.2–6.4%)	
5	5	3.5% (2.50)		3.8% (1.7–7.8%)	
Semester					
1	10	1.7% (2.33)		–	–
2	10	2.2% (2.23)	0.564	–	
Annual	10	2.8% (2.39)		–	
Alternate test form					
No	6	3.8% (2.07)	0.136	–	–
Yes	24	1.9% (2.29)		–	
Length of examination					
≤ 50 items	15	1.4% (2.21)	0.020	1.3% (0.7–2.4%)	0.038
> 50 items	15	3.4% (2.08)		3.3% (1.9–5.4%)	
Number of options					
< 5 options	6	3.4% (1.59)	0.244	–	–
≥ 5 options	24	1.9% (2.42)		–	
Mean percentage of correct answers					
< 66%	14	1.8% (2.31)	0.380	–	–
≥ 66%	16	2.5% (2.28)		–	
Mean percentage of missing answers					
< 5%	24	1.8% (2.39)	0.004	–	–
≥ 5%	6	4.1% (1.29)		–	

*Abbreviations*: *CI* Confidence interval, *GM* Geometric mean, *GSD* Geometric standard deviation

^a^Adjusted for year and length of examination

The year of medical school and the length of examination were associated with an increase of anomalies detected. Both associations were significant, with a *p*-value of 0.049 and 0.020, respectively. Examinations where alternate test forms were used had a geometric mean of anomalies of 1.9%, and in those where they were not used, the geometric mean of anomalies was 3.8%. The use of alternate test forms was associated with a decrease in anomalies; however, this association was not significant (*p* = 0.136). The semester had very little impact on the amount of anomalies detected.

**Fig. 2:**
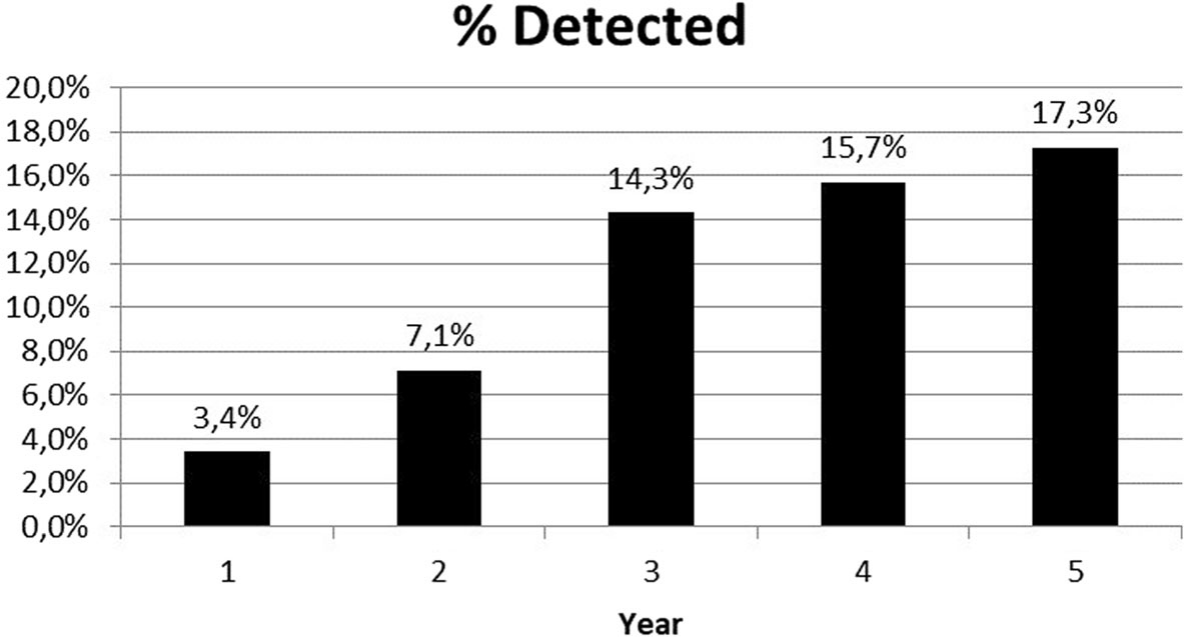
Percentage of students detected in anomaly/cheating pairs by year of medical school

The prevalence of students with anomalies detected in at least one course in a year of medical school is represented in Fig. 2. Approximately 13% (11.7–15.2 95%CI) of students were detected anomalies at least once. Anomalies were lowest in the first year with 3.4% (1.4–5.3 95%CI) students detected and highest in the fifth year, with 17.3% (13.4–21.2 95%CI). Anomalies increased with the year of medical school.

### Social networks in anomalies/cheating

**Fig. 3:**
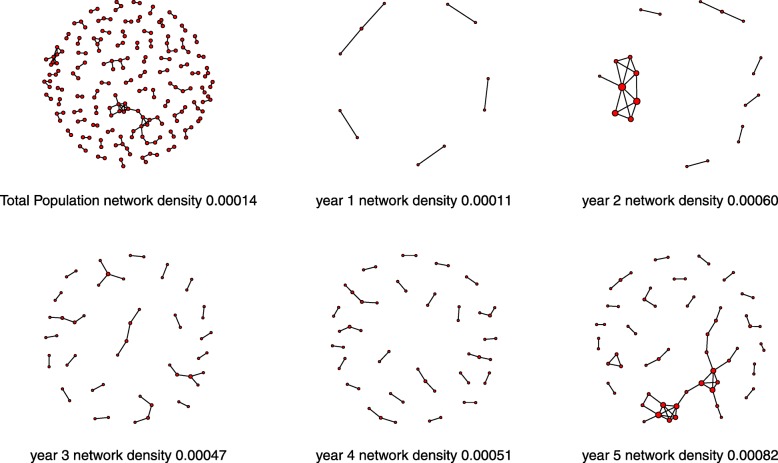
Social network of anomalies/cheating by means of copying answers by year of medical school. Vertices without connections (students not detected) are not represented

Analysis of the networks is represented in Fig. 3. A total of 151 anomaly pairs (edges) were detected, composed of 200 students (vertices). Of these, 25% were involved in more than one anomaly pair. The highest amount of times a student was identified in a different course was eight. The density of the social network was lowest in the first year and highest in the fifth year, with 1.12E-04 and 8.20E-04, respectively. We identified six cliques: two with three members, three with four members and one with five members.

## Discussion

The AP refers to the percentage of anomalies/cheating detected in written examination; the geometric mean prevalence was 2.18%. Just over 150 anomaly pairs from a total of 7403 examinees were detected, which is considerably higher than the findings of McManus and colleagues in postgraduate students, where only 13 anomaly pairs were found among 11,518 examinees [23]. However, McManus et al. used a statistical significance level of 0.001, while we used a statistical significance level of 0.05, so fewer cases would be found compared to those of the present study. Further, the exams analysed by McManus et al. had more items, and hence a greater power for detecting anomalies, so many more cases would be identified compared to our study. Nevertheless using the same significance level as McManus, we obtained 22 anomaly pairs, which is still a higher number compared to their findings. Although we used the Šidák correction to minimize type error I, the number of pairs detected would be the same if we had used the Bonferroni correction.

However, the AP refers to the percentage of detected individuals, which includes false positives and true positives, and excludes false negatives. The TP of anomalies/cheating in an examination was estimated to be 1.85% when adjusted to the SEN and SPE of the method. To our knowledge, this is the first study that tries to estimate the TP of anomalies on written examinations using statistical methods; previous studies have only reported the AP.

When considering anomalies at least once in a year, 13.4% of the students were involved in anomalies. Our findings reveal a higher prevalence of anomalies at least once in a written examination than those reported by Dyrbye and colleagues (2010) [9], and Rennie and Crosby (2001) [12], but lower than Genereux and McLeod (1995) [13], Hrabak and colleagues (2004) [14], Hafeez and colleagues (2013) [15] and Sivagnanam and colleagues (2002) [16]. Our findings also reveal a prevalence of anomalies/cheating considerably lower than the findings of Teixeira et al., who also conducted their study in Portugal [30].

We have performed a simulation study in order to estimate the SEN and SPE necessary to estimate the TP. The results of the simulated data are shown in Additional file 1: Table S2. By correcting alpha levels using the Šidák correction, SPE is always near 100% and independent of the amount of cheating simulated. As for SEN, it increases with the amount of cheating simulated. Detection rates are higher for all percentages of copied items when the examination has 100 items when compared to 50 items. This is most likely the reason for finding a significant association between anomalies by means of copying answers and the length of the examination. When calculating the other factors that may influence anomalies and the TP, we adjusted for the effect of the examination’s length, so this flaw in the statistical method used should not affect other findings in our study.

The major limitation when using statistical methods that analyze similarity between responses is that the similarity may result from factors other than cheating. Studies have shown that friendship has an impact on students’ grades [4]. The most common argument presented is that students who study together may present a very similar answer pattern, and be wrongly accused of cheating. However, in previous studies, it has been pointed out that most of the anomaly pairs detected through statistical methods were in an adjacent seat [31]. McManus and colleagues applied the Acinonyx on a nationally administered examination in the United Kingdom, and when seating plans were available, all anomaly pairs detected were sitting side by side [23]. A large number of students work together, but there are usually very limited opportunities for adjacent seating. In our case, the general rule in our faculty of seating is by alphabetical order of the given name and consequently it is possible to collude in advance. Nevertheless, we would assume that students who study together would do so for the majority of the courses. Thus, if the answer similarity detected was the result of students studying together; the percentage of detected students would be very similar in all courses for that year. That was not the case; we found some variability within each year of medical school.

The only variable that was significantly associated with anomalies is the year of medical school. The percentage of students involved in anomalies at least once in a year was the lowest in the first and highest in the fifth, increasing with year of medical school. It seems that the longer students stay in medical school, the more prone to anomalies they become. This result is similar to that of Hrabak et al. [14].

To find an explanation, we created networks from the cheating/anomaly pairs found. At a first glance, we found that students seem to aggregate and create clusters. Students who cheat in many courses cheat from others who are themselves involved in more than one cheating/anomaly pair. Analyzing the network, we also found that the density is lowest in the first year and highest in the fifth. It is clear that students create social networks of cheating/anomalies that grow as medical school progresses. To our knowledge, this is the first time that the presence of social networks in cheating/anomalies has been documented.

The increase of anomalies with year of medical school can be explained by the formation of social networks of learning or revision, and that of course has a rather more benign explanation, since we probably want students to study with their peers. Indeed, it can be encouraged by participation in collaborative projects, which are a necessary preparation for life in the real world. However, the formation of social networks could be interpreted as a challenge to those involved in assessment and we should take measures to prevent anomalies/cheating within close social groups but also set examination challenges that test team ability.

The limitations of our last findings was the number of confounders taken into account, we only included the year of medical school, semester and length of examination, other variables such as the number of instructors proctoring or the number of students per room should be considered and this analysis was tested at the level of examinations (*n* = 30) and consequently there is little statistical power.

Another limitation is that the Angoff *A* method does not use distractors to detect similarity between the answers assuming that the distractors in the MCQ’s are equally difficult, items that have distractor with different level of difficulty will be identified as more likely to be cheated over. However, we believe that the effect on the TP is small considering that we corrected for SEN and SPE of the method, and the effect on the social network would be the underestimation of the size of the network.

## Conclusions

Overall the study of social networks between anomaly/cheating pairs will allow governing bodies (e.g. medical registration boards) to established the foundations for professional/unprofessional behavior during medical training, and provides a new perspective on cheating as a group-based rather than individual activity, which expands on the existing literature and provides interesting insight into the social structure around cheating and when (i.e. increasingly with year of education) medical students are most likely to cheat.

Applying these methods are an effective and easy way to assess the severity of this problem in any school, and can help determine which measures should be applied and to evaluate their effectiveness. As an example, a new guideline should be adopted where students sit according to a random seating plan, with a new plan for each exam; otherwise students know who they will be sitting next to.

It would also be interesting to apply a statistical method and social networks of cheating in a longitudinal study, and to cross these with the formation and evolution of social networks of teaching, learning or revision.

## Additional file

Additional file 1: **Table S1.** Results for apparent prevalence and true prevalence by course. **Table S2.** Results for 50 simulations of each percentage of copied items for examinations with 50 and 100 items. Table S1 shows the distribution of anomalies among all courses included in this study. In Table S2 are shown the results of the simulated study performed to estimate the SEN and SPE necessary to estimate the TP. (DOCX 25 kb)

## Abbreviations

AP: Apparent prevalence
CES: Centro Hospitalar São João Ethics Committee for Health
CI: Confidence Interval
FMUP: Faculty of Medicine of the University of Porto
GBT: Generalized binominal test
GM: Geometric mean
GSD: Geometric standard deviation
SEN: Sensitivity
SPE: Specificity
TP: True prevalence
US: United States
