# Supplementary material for: Investigating the existence of social networks in cheating behaviors in medical students
Source: BMC Med Educ. 2018 Aug 9;18:193. doi: 10.1186/s12909-018-1299-7 (PMC6085665; doi:10.1186/s12909-018-1299-7)
Supplement: Supplementary file 1 — Table S1. Results for apparent prevalence and true prevalence by course. Table S2. Results for 50 simulations of each percentage of copied items for examinations with 50 and 100 items. Table S1 shows the distribution of anomalies among all courses included in this study. In Table S2 are shown the results of the simulated study performed to estimate the SEN and SPE necessary to estimate the TP. (DOCX 25 kb) [file 12909_2018_1299_MOESM1_ESM.docx]

Additional file 1

Table S1. Results for apparent prevalence and true prevalence by course

| Year | Course | ATF | LOE | Examinees | Detected | AP | TP |
| --- | --- | --- | --- | --- | --- | --- | --- |
| 1^st^ |  |  |  |  |  | % | % |
|  | 1 | Yes | 62 | 267 | 10 | 3.7 | 4.7 |
|  | 2 | Yes | 50 | 249 | 2 | 0.8 | 0.7 |
|  | 3 | Yes | 25 | 254 | 0 | 0.0 | 0.1 |
|  | 4 | Yes | 34 | 241 | 0 | 0.0 | 0.1 |
|  | 5 | Yes | 65 | 262 | 0 | 0.0 | 0.1 |
| 2^nd^ |  |  |  |  |  |  |  |
|  | 1 | Yes | 42 | 196 | 11 | 5.6 | 7.8 |
|  | 2 | Yes | 54 | 215 | 9 | 4.2 | 5.4 |
|  | 3 | Yes | 42 | 169 | 4 | 2.4 | 3.0 |
|  | 4 | Yes | 50 | 218 | 4 | 1.8 | 2.1 |
|  | 5 | Yes | 52 | 233 | 4 | 1.7 | 2.3 |
|  | 6 | Yes | 54 | 262 | 2 | 0.8 | 0.4 |
|  | 7 | Yes | 34 | 126 | 0 | 0.0 | 0.3 |
|  | 8 | Yes | 28 | 230 | 0 | 0.0 | 0.1 |
| 3^rd^ |  |  |  |  |  |  |  |
|  | 1 | Yes | 190 | 183 | 11 | 6.0 | 5.6 |
|  | 2 | Yes | 150 | 215 | 12 | 5.6 | 5.2 |
|  | 3 | Yes | 125 | 183 | 8 | 4.4 | 4.1 |
|  | 4 | No | 110 | 283 | 10 | 3.5 | 3.2 |
|  | 5 | Yes | 190 | 261 | 8 | 3.1 | 2.8 |
|  | 6 | No | 110 | 263 | 8 | 3.0 | 2.9 |
|  | 7 | Yes | 100 | 220 | 4 | 1.8 | 1.5 |
| 4^th^ |  |  |  |  |  |  |  |
|  | 1 | No | 80 | 287 | 19 | 6.6 | 6.4 |
|  | 2 | Yes | 50 | 313 | 14 | 4.5 | 5.8 |
|  | 3 | No | 50 | 325 | 13 | 4.0 | 5.0 |
|  | 4 | Yes | 50 | 267 | 7 | 2.6 | 3.1 |
|  | 5 | Yes | 28 | 252 | 6 | 2.4 | 2.9 |
| 5^th^ |  |  |  |  |  |  |  |
|  | 1 | No | 60 | 308 | 34 | 11.0 | 13.6 |
|  | 2 | Yes | 63 | 246 | 22 | 8.9 | 11.6 |
|  | 3 | Yes | 40 | 288 | 8 | 2.8 | 3.6 |
|  | 4 | Yes | 45 | 291 | 5 | 1.7 | 1.8 |
|  | 5 | No | 40 | 296 | 2 | 0.7 | 0.6 |
| Geometric Mean (95CI) | | | | | | 2.18 | 1.85 |
|  |  |  |  |  |  | (1.43-3.19) | (1.07-3.20) |

Abbreviations: ATF – Whether alternate test forms were used or not. LOE – length of examination. AP – Apparent prevalence. TP – True prevalence

Table S2. Results for 50 simulations of each percentage of copied items for examinations with 50 and 100 items.

|  | Percentage copied | 50 | 55 | 60 | 65 | 70 | 75 | 80 | 85 | 90 | 95 | 100 |
| --- | --- | --- | --- | --- | --- | --- | --- | --- | --- | --- | --- | --- |
| 50 Items |  |  |  |  |  |  |  |  |  |  |  |  |
|  | Sensitivity | 2 | 4 | 10 | 14 | 32 | 47 | 68 | 80 | 91 | 94 | 98 |
|  | Specificity | 100 | 100 | 100 | 100 | 100 | 100 | 100 | 100 | 100 | 100 | 100 |
| 100 Items |  |  |  |  |  |  |  |  |  |  |  |  |
|  | Sensitivity | 39 | 59 | 80 | 91 | 97 | 99 | 100 | 100 | 100 | 100 | 100 |
|  | Specificity | 100 | 100 | 100 | 100 | 100 | 100 | 100 | 100 | 100 | 100 | 100 |
